# BRCA1-Dependent Translational Regulation in Breast Cancer Cells

**DOI:** 10.1371/journal.pone.0067313

**Published:** 2013-06-21

**Authors:** Estelle Dacheux, Anne Vincent, Nicolas Nazaret, Christophe Combet, Anne Wierinckx, Sylvie Mazoyer, Jean-Jacques Diaz, Joël Lachuer, Nicole Dalla Venezia

**Affiliations:** 1 Université de Lyon, Lyon, France; 2 Université Lyon 1, ISPB, Lyon, France; 3 INSERM U1052, Centre de Recherche en Cancérologie de Lyon, Lyon, France; 4 CNRS UMR5286, Centre de Recherche en Cancérologie de Lyon, Lyon, France; 5 Equipe Labellisée LIGUE 2008, Lyon, France; 6 ProfileXpert, Lyon, France; 7 CNRS UMR5086, Unité Bases Moléculaires et Structurales des Systèmes Infectieux, Lyon, France; National Cancer Institute, United States of America

## Abstract

BRCA1 (Breast Cancer 1) has been implicated in a number of cellular processes, including transcription regulation, DNA damage repair and protein ubiquitination. We previously demonstrated that BRCA1 interacts with PABP1 (Poly(A)-Binding Protein 1) and that BRCA1 modulates protein synthesis through this interaction. To identify the mRNAs that are translationally regulated by BRCA1, we used a microarray analysis of polysome-bound mRNAs in BRCA1-depleted and non-depleted MCF7 cells. Our findings show that BRCA1 modifies the translational efficiency of approximately 7% of the mRNAs expressed in these cells. Further analysis revealed that several processes contributing to cell surveillance such as cell cycle arrest, cell death, cellular growth and proliferation, DNA repair and gene expression, are largely enriched for the mRNAs whose translation is impacted by BRCA1. The BRCA1-dependent translation of these species of mRNAs therefore uncovers a novel mechanism through which BRCA1 exerts its onco-suppressive role. In addition, the BRCA1-dependent translation of mRNAs participating in unexpected functions such as cellular movement, nucleic acid metabolism or protein trafficking is indicative of novel functions for BRCA1. Finally, this study contributes to the identification of several markers associated with BRCA1 deficiency and to the discovery of new potential anti-neoplastic therapeutic targets.

## Introduction

Loss of function of the tumour suppressor BRCA1 (Breast Cancer 1) protein is responsible for numerous familial and sporadic breast cancers. *BRCA1* inactivating mutations are found in 8–10% of patients with familial breast cancer [1]. In contrast, *BRCA1* mutations are rare in sporadic cancers [2]. Nevertheless, sporadic breast cancers represent up to 90% of breast cancers and are often characterized by decreased *BRCA1* expression at mRNA and protein levels [3] [4] [5].

The BRCA1 protein is involved in a number of cellular processes, including regulation of transcription, DNA repair, cell cycle checkpoint, protein ubiquitination and apoptosis [6]. Furthermore, BRCA1 associates with several proteins involved in these functions. For example, BRCA1 interacts with the RNA polymerase II holoenzyme complex in part through binding to RNA helicase A, and also interacts with the repair protein RAD51 or the ubiquitin ligase BARD1. The capacity of BRCA1 to form multiple protein complexes contributes to its central role in cell surveillance. However, further studies are needed to fully appreciate the molecular mechanisms underlying the role of BRCA1 as a tumour suppressor.

BRCA1 is a nucleo-cytoplasmic shuttling protein containing two nuclear localization sequences and two nuclear export sequences. BRCA1 shuttling is regulated *via* different types of protein-protein interaction [7]. Recent studies suggest that control of BRCA1 sub-cellular localization is a vital process in the regulation of BRCA1 functions [8] [9] [10]. BRCA1 shuttling may not only control its nuclear function in DNA repair but might also facilitate additional cellular processes involved in the execution of DNA damage-induced cell death [11] [12] [13]. The dynamic equilibrium between BRCA1 nuclear import and export provides a new level of regulation of its nuclear and cytoplasmic functions.

We previously identified PABP1 as a novel BRCA1 partner and showed that BRCA1 modulates translation through its interaction with PABP1 in the cytoplasm. We showed that the global translation was diminished in BRCA1-depleted cells and increased in BRCA1-overexpressing cells [14]. Because BRCA1 is clearly not a canonical factor of translation, our findings suggested that the effect on global translation results from the targeting of a subset of mRNAs by BRCA1, rather than an effect on all cellular mRNAs. Because BRCA1 is a tumour suppressor, a number of mRNAs translationally regulated by a BRCA1-dependent mechanism may contribute to cell surveillance by encoding DNA repair factors, cell cycle and cell death regulators or transcription factors.

In the present study, we have identified which mRNAs are translationally regulated by BRCA1 using a microarray analysis of polysome-associated RNAs from BRCA1-depleted MCF7 cells. Our findings confirm our hypothesis that BRCA1 affects translation of a subset of mRNAs. These findings allow the proposition that any of these translated products contribute to the BRCA1 tumour suppressor activity.

## Materials and Methods

### Chemicals

The primary antibodies used in this study were as follows: mouse monoclonal antibodies to BRCA1 (8F7 (GeneTex), D9 (Santa Cruz) and MS110 (Calbiochem)), TOP1 (C21 (BD Biosciences)), PABP1 (10E10 (Sigma)), β-Actin (AC-15 (Sigma)) and α-Tubulin (DM1A) (Calbiochem), rabbit polyclonal antibodies to eIF4G (H-300 (Santa Cruz)) and HIPK2 (Aviva), rabbit monoclonal antibodies to HIPK2 (Epitomics). Secondary antibodies used were peroxydase-conjugated anti-mouse or anti-rabbit immunoglobulins (Jackson ImmunoResearch).

### Cell Culture and Transfection

The human epithelial mammary cell line MCF7 that is available from ATCC was maintained in DMEM containing 4.5 g/l of glucose supplemented with 0.1 mM non-essential amino acids, 1 mM sodium-pyruvate, 0.01 mg/ml recombinant human insulin, 10% foetal calf serum, 100 µg/ml streptomycin and 100 units/ml of penicillin.

The human embryonic kidney cell line 293T that is available from ATCC was maintained in DMEM containing 4.5 g/l of glucose supplemented with 10% foetal calf serum, 100 µg/ml streptomycin and 100 units/ml of penicillin.

The siRNA duplexes were purchased from MWG (Germany) and provided as purified and annealed duplexes. The sequences of the siRNAs against BRCA1 were Si-BRCA1 5′-GGAACCUGUCUCCACAAAG-3′ and Si-control 5′-CACGAUGUGACAGUGAUAU-3′ [14]. For transfection, cells were plated at 1.8×10^6^ cells per 10 cm diameter dish 24 h before transfection. Cells were transfected with 200 pmol/dish of siRNA and 16 µl/dish of Lipofectamine 2000 (Life Technologies) using the protocol of the supplier. Cells were harvested 72 hours after transfection.

The pCDNA3β-BRCA1 plasmid expressing BRCA1 full length protein was previously described [15]. Cells were plated at 4×10^6^ cells per 10 cm diameter dish 24 h before transfection. Cells were transfected with 4 µg of BRCA1 expressing vector and 20 µl of ExGen 500 (Euromedex) following the supplier procedure. Twenty-four hours after transfection, cells were harvested.

### Ribosome Purification

This procedure was performed essentially as described before [16]. Extracts from MCF7 cells were prepared by lysis at 4°C in extraction buffer (50 mM Tris-HCl, pH 7.4, 25 mM KCl, 5 mM MgCl_2_, 250 mM sucrose, 0.7% Nonidet-P40) and nuclei were removed by centrifugation (800 g, 10 min, 4°C). The supernatant was centrifuged (12 000 g, 10 min, 4°C) to eliminate mitochondria. The supernatant (cytoplasmic fraction) was layered onto 1 ml of a 1 M sucrose cushion made in 50 mM Tris-HCl, pH 7.4, 25 mM KCl, 5 mM MgCl_2_, and centrifuged for 2 h at 250 000 g and at 4°C in a TL100 rotor (Beckman). The ribosome pellet was resuspended in buffer (50 mM Tris-HCl, pH 7.4, 25 mM KCl, 5 mM MgCl_2_). Aliquots of nuclear and cytoplasmic fractions and the resuspended ribosome pellet were then submitted to further immunoblot analysis.

### Immunoblotting

Cells were lysed in lysis buffer A (20 mM Hepes-KOH pH 7.2, 100 mM KCl, 1 mM DTT, 0.5 mM EDTA, 0.5% NP40, 10% Glycerol) supplemented with protease inhibitor (Complete EDTA free, Roche). Protein concentrations were determined by Bradford procedure (BioRad). Proteins were subjected to SDS-PAGE, and blotted onto poly-vinylidene difluoride (PVDF) membranes (Immobilon-P, Millipore). Membranes were blocked in Tris-buffered saline solution containing 0.05% Tween 20 and 5% non fat milk and incubated with primary antibodies. Horseradish peroxidase conjugated secondary antibodies (Jackson ImmunoResearch) were used for detection of immunoreactive proteins by chemiluminescence (ECL, GE Healthcare).

### Isolation of Polysomes and Total Cytoplasmic RNA

Extracts from MCF7 cells were prepared as described in the “Ribosome purification” section. Then, the supernatant containing the cytoplasmic fraction was divided in two portions. One fifth of the cytoplasmic fraction was used as a source for total cytoplasmic RNA (total RNA) using Trizol (Life Technologies) extraction protocol and isopropanol precipitation. The remaining 80% of the cytoplasmic fraction was layered onto an 11 ml linear sucrose gradient (10–40% sucrose supplemented with 50 mM Tris-HCl, pH 7.4, 25 mM KCl, 5 mM MgCl_2_, 10 mM DTE and 100 µg/ml cycloheximide). A 2 h centrifugation at 250 000 g and at 4°C in a SW41Ti rotor (Beckman) was performed.

To construct the ribosome profile, 36 fractions of 300 µl were manually collected after centrifugation. RNA from each fraction was recovered by Trizol extraction protocol and isopropanol precipitation. RNA was resuspended in 20 µl of RNase-free water and quantified by absorbance at 260 nm using a Nanodrop. A graph was drawn to visualize the variation of RNA concentration as a function of the fraction and thus determine which fractions contained monosomal or polysomal material. For agarose gels, RNA recovered from the 36 fractions was pooled so as to obtain 18 fractions and the fractions were resolved on a 1% agarose gel. For protein analysis, 18 fractions of 600 µl were manually collected. Proteins were precipitated by trichloroacetic acid (TCA) to a final concentration of 20% and washed with ice-cold acetone. Proteins were resolved by SDS-PAGE and immunoblotted.

For microarray analysis, 18 fractions of 600 µl were manually collected. RNA was recovered from individual fractions by Trizol extraction and isopropanol precipitation. RNA from the polysomes (fractions 12 to 18) was pooled (polysomal RNA). Therefore, in these experimental conditions, total cytoplasmic RNA and polysomal RNA originate from a common cytoplasmic fraction.

### Microarray Analysis

Total cytoplasmic RNA and polysomal RNA were isolated from BRCA1-depleted MCF7 cells and control MCF7 cells as described in the “Isolation of polysomes and total cytoplasmic RNA” section.

Microarray processing and data analysis was performed at the ProfileXpert core facility (Lyon, France). Microarray analysis was performed using a high-density oligonucleotide array (GeneChip Human Genome U133 Plus 2.0 array, Affymetrix, Santa Clara, CA, USA). RNA (100 ng) was amplified and biotin-labeled using GeneChip® 3′ IVT Express target labelling and control reagents and procedures from Affymetrix. Before amplification, spikes of synthetic mRNA at different concentrations were added to all samples; these positive controls were used to ascertain the quality of the process. Biotinylated antisense cRNA for microarray hybridization was prepared. After final purification using magnetic beads, cRNA quantification was performed with a nanodrop and quality checked with the Agilent 2100 Bioanalyzer (Agilent technologies, Inc, Palto Alto, CA, USA).

Hybridization was performed following Affymetrix protocols (http://www.affymetrix.com). Briefly, labelled cRNA was fragmented and denatured in hybridization buffer, then 10 µg were hybridized on the chip for 16 h at 45°C with constant mixing by rotation at 60 rpm in an Genechip hybridization oven 640 (Affymetrix). After hybridization, arrays were washed and stained with streptavidin-phycoerythrin (GeneChip® Hybridization Wash and Stain Kit, Affymetrix) in the Fluidics Station 450 (Affymetrix) according to the manufacturer’s instructions. The arrays were read with a confocal laser (GeneChip® Scanner 3000 7G, Affymetrix). Then CEL files were generated using the Affymetrix GeneChip Command Console (AGCC) software 3.0.

The complete set of raw and normalized data is available at the GEO database under accession number GSE40730.

### Data Filtering and Analysis

The obtained data were normalized with Affymetrix Expression Console software using the MAS5 statistical algorithm. Normalized data were compared and filtered using Partek Genomic Suite software 6.5 (Partek Inc., St. Louis, MO, US).

First, for each RNA type (polysomal RNA or total RNA prepared from the same cytoplasmic extract as described in the “Isolation of polysomes and total cytoplasmic RNA” section), samples were paired by replicate and a ratio between siBRCA1 and control sample was calculated. The ratios for the polysomal RNA (polyRNA = siBRCA1/siControl) and for total RNA (totRNA = siBRCA1/siControl) were calculated. A gene was considered only if the detected signal was above the background for at least one of the compared groups.

Then, for each retained gene and for each replicate a polyRNA/totRNA Ratio (Ratio of Ratio = RR) was calculated as follows:

RR_i_ = polyRNA_i_/totRNA_i_ (i = replicate).

Only genes showing a polyRNA/totRNA Ratio greater than 1.5 or lower than 0.67 in the two replicates were retained. The retained genes of interest were listed and classified according to their biological functions using Ingenuity Pathways Analysis (IPA) (Ingenuity Systems Inc., Redwood City, California).

### Quantitative RT-PCR

Prior to reverse transcription, the total and polysomal RNA described above from BRCA1-depleted and control cells were purified and submitted to DNase digestion with NucleoSpin RNA XS Clean-up columns (Macherey-Nagel) following the manufacturer’s instructions. Reverse transcription was then performed using 500 ng of total RNA, with Bio-Rad Laboratories’ iScript cDNA Synthesis Kit in a total volume of 20 µl. The reaction was incubated at 25°C for 5 min followed by incubation at 42°C for 30 min and 85°C for 5 min. Quantitative real-time PCR was performed as follows. The amount of cDNA synthesized was measured using q-PCR (SYBR Green PCR, LightCycler, Roche Diagnostics Indianapolis) following the manufacturer’s recommendations. The LightCycler experimental run protocol consisted in an initial Taq activation for 8 min at 95°C, followed by a “touch down” program. The “touch down” program consisted of 15 s at 95°C, 5 s at 68°C, and 8 s at 72°C followed by a progressive decrease of the annealing temperature which was reduced by 0.5°C every successive cycle until 62°C (13 cycles). A generic PCR amplification of up to 27 cycles was then performed using the final annealing temperature reached in the “touch down” phase. A melting curve step was used to examine each sample for purity. Quantitative PCR analysis was performed in triplicate.

Primers were designed with Primer-Blast software (National Centre for Biotechnology Information/NCBI, Bethesda, USA) and purchased from MWG (Germany). All primers had Tms between 59 and 61°C and all the products were 100–200 bp. The primers used were as follows: HPRT1 (forward, 5′-TCTGTGGCCATCTGCTTAGTAGAGC-3′; reverse, 5′-ACAATCCGCCCAAAGGGAACTGA-3′), FAM110B (forward, 5′-GCGCAAGCATGATCAGCTCAGAC-3′; reverse, 5′-TGGCAGAAATGCCATACGGCAC-3′), HIPK2 (forward, 5′-ACAAGACATTCCAGCCCCAGGGA-3′; reverse, 5′-CACAGACAGGGAATGAAGCCTGCA-3′), SMC6 (forward, 5′-TCTTTCCCTGTGGTCCATCGCAGA-3′; reverse, 5′-ACGCTGGGAATCTGCCATCTTCAG-3′), THRA (forward, 5′-ATGCCCTCAACTCACCCCCTACA-3′; reverse, 5′-CCAAGCCAAGCCGTTCTTTGCAC-3′) and TOP1 (forward, 5′-ATGAGTGGGGACCACCTCCACA-3′; reverse, 5′-CGGAAATCCGCTTCGATCTGGGA-3′).

Relative quantification was carried out using the LightCycler software (version 4.1). The levels of the housekeeping gene hypoxanthine guanine phosphoribosyl transferase 1 (HPRT1) transcript were used to normalize the potential amount variation of sample cDNAs added to each reaction. For each gene, the polyRNA and the totRNA Ratios were then determined using the ΔΔCt calculation method.

### Statistical Analysis

Data are reported as mean ± standard error of mean (SEM). Statistical significance was determined using two-tailed paired Student’s *t* test and *P* values of less than 0.05 were considered significant.

## Results

### BRCA1 is a Ribosome-associated Protein

**Figure 1:**
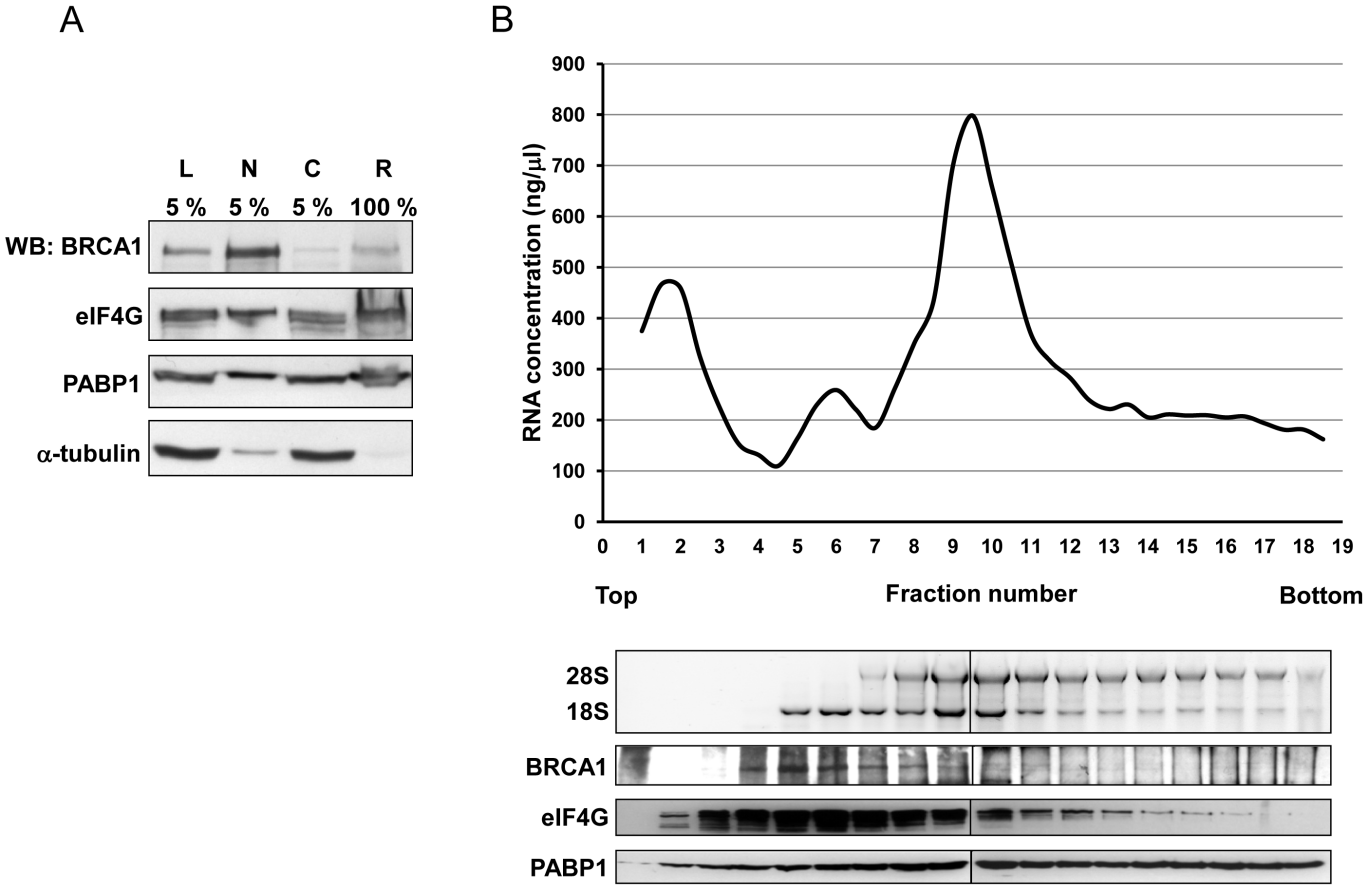
BRCA1 is a ribosome-associated protein. A/MCF7 cells were lysed in 25 mM KCl buffer and the post-mitochondrial cytoplasmic lysate was layered onto a 1 M sucrose cushion and centrifuged as described in the “Material and methods” section. Immunoblotting for BRCA1 using the MS110 antibody was performed on the following samples: initial total cell lysate (L), nuclear fraction (N), cytoplasmic fraction (C) and ribosome pellet (R). PABP1 and eIF4G were used as markers for pellet fraction containing ribosome-associated proteins. The analyzed L, N and C fractions represent 5% of the total cell lysate. B/MCF7 cells were lysed in 25 mM KCl buffer and the cytoplasmic fraction was separated onto a 10–40% sucrose gradient. (Top) A characteristic ribosome profile. (Middle) Extracts of total RNA from half of each fraction were subjected to gel analysis to determine the presence of 18S and 28S rRNAs. rRNAs were detected by Gel Red staining. (Bottom) The remaining half of each fraction was precipitated with TCA. BRCA1 protein was identified with immunoblot analysis using D9 antibody. PABP1 and eIF4G served as controls.

Because BRCA1 stimulates translation through its interaction with PABP1 [14], we investigated whether BRCA1 is associated with ribosomes. MCF7 cells were fractionated and total cell lysate, nuclear, cytoplasmic and ribosomal fractions purified in low monovalent cation concentration (25 mM KCl) were analyzed for the presence of BRCA1 by immunoblotting (Figure 1A). As expected because BRCA1 is known to be mainly nuclear, the amount in the nuclear fraction was more important than that in the cytoplasmic fraction. Nevertheless, a large part of the cytoplasmic BRCA1 pool was recovered in the ribosomal fraction. In low monovalent cation concentrations, ribosomal proteins and non-ribosomal proteins that are associated with ribosomes as part of the translational machinery, such as PABP1 and different eukaryotic translation initiation and elongation factors (eIF and eEF) are purified [17]. To further ascertain that the ribosomal fraction purified in these conditions contained some factors of the translational machinery, we analyzed by immunoblotting the presence of eIF4G and PABP1 in this fraction. As shown in figure 1A, the ribosomal fraction contained both proteins. In addition, the specificity of the fractionation procedure was reinforced by the fact that α-tubulin, a cytoskeleton protein, was not found concentrated within this ribosomal fraction. Therefore, BRCA1 was found to be associated with the ribosomal fraction of the cells.

To reinforce this conclusion and to determine whether BRCA1 is associated preferentially to free ribosomal subunits or to monosomal or polysomal complexes, the cytoplasmic fractions were fractionated by sucrose density gradients. A graph was drawn to visualize the variation of RNA concentration as a function of the fraction and thus determine which fractions contained free ribosomal subunits, monosomal or polysomal material (Figure 1B). RNA recovered so as to obtain 18 fractions was analyzed through a 1% agarose gel to determine the presence of 18S and 28S rRNA. In parallel, each fraction was analyzed by western blotting to determine the presence of BRCA1. The majority of BRCA1 was detected in fractions 4 to 12, therefore mainly in sub-polysomal fractions that contain 40S, 60S and 80S ribosome subunits (Figure 1B). Western blotting to detect PABP1 and eIF4G were performed as controls. As previously shown, PABP1 was found with sub-polysomal and polysomal fractions [18] whereas eIF4G was found mainly with sub-polysomal fractions [19].

Taken together, these results indicate that BRCA1 is a ribosome-associated protein that is recruited to the translation apparatus similarly to the translation initiation factor eIF4G.

### Microarray Analysis Shows that a Subset of mRNAs is Subject to Differential Translational Regulation Following BRCA1 Depletion

To identify translational targets of BRCA1, we performed a microarray analysis of polysome-associated mRNA and total cytoplasmic mRNA from BRCA1-depleted and non-depleted MCF7 cells.

**Figure 2:**
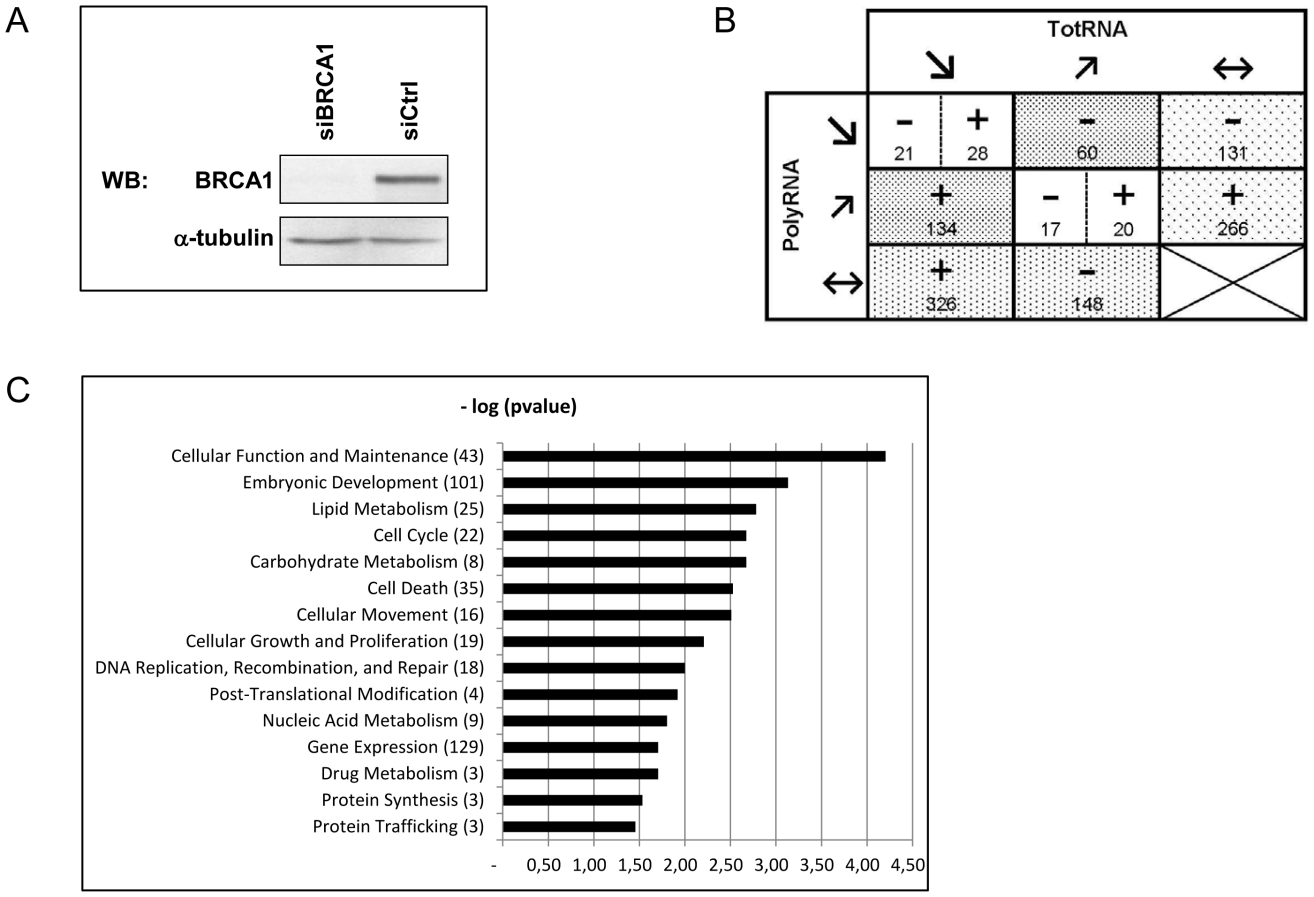
Microarray analysis of polysome-associated RNAs from MCF7 cells in which BRCA1 has been depleted. A/Western blot confirming siRNA inhibition of BRCA1 levels in MCF7 cells when compared with control siRNA. Immunoblotting for BRCA1 used 8F7 antibody. α-tubulin served as loading control. B/Number of mRNAs exhibiting altered translational efficiency in BRCA1-depleted MCF7 cells compared to control MCF7 cells. The 1151 mRNAs displaying a modified relative translatability (RR = polyRNA/totRNA) were clustered in several groups depending on their fold change in polysomal RNA abundance (PolyRNA) and their fold change in total mRNA abundance (TotRNA). The fold changes in polysomal RNA abundance and in total mRNA abundance are indicated as follows: 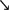 ≤0.67, (↗)≥1.50, (↔) >0.67 and <1.50. The RRs are annotated with a sign and a number. The sign specifies the RR value: (−) ≤0.67, (+) ≥1.50. The number indicates how many mRNAs are deregulated. 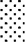 : mRNAs translationally deregulated through change in polysome mRNA abundance only; 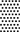 : mRNAs translationally deregulated through change in total mRNA abundance only; 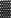 : mRNAs translationally deregulated through change in polysome abundance together with opposite changes in total mRNA C/Functional distribution of differentially translated known genes in BRCA1-depleted versus control MCF7 cells. Gene functions were established based on the annotation provided by the IPA database. The number of genes enriched in each function is shown in brackets.

BRCA1 was depleted in two independent experiments using the previously described BRCA1-targetting siRNA [20] [14]. Control MCF7 cells were transfected with the control siRNA as previously described. BRCA1 depletion was confirmed at the protein level prior to polysome and RNA isolations (Figure 2A). The microarray analyses were performed from these two independent experiments using GeneChip Human Genome U133 Plus 2.0 array (Affymetrix).

Translation being a cytoplasmic event, we focused the analyses on the mRNAs isolated from the cytoplasmic cellular fraction. As described in the “Material and methods” section, we used one fifth of this fraction as a source of total cytoplasmic mRNA (totRNA) and the mRNAs associated to polysomes (fractions 12 to 18) (polyRNA) were isolated from the rest of this fraction.

We first investigated the variations in mRNA content within the totRNA fraction to determine whether BRCA1 modulates the amounts of cytoplasmic mRNAs reflecting their rates of synthesis, transport and stability. Among the 17416 mRNAs expressed in MCF7 cells, 820 displayed modified cytoplasmic abundance in BRCA1-depleted cells as compared to non-depleted cells. This change was therefore conferred by the absence of BRCA1, and concerned all cytoplasmic mRNAs, whatever their intra-cytoplasmic localization (sub-polysomes, polysomes, P-bodies,…).

We then investigated whether BRCA1 modulates the translational efficiency of the cytoplasmic mRNAs as reflected by their distribution within the polysomal fraction. To evaluate how BRCA1 affects mRNA association with polysomes, we analyzed the amount of polysomal mRNA both in presence and absence of BRCA1. We determined the relative translatability of each mRNA by calculating the following ratio: change in abundance in polysomal mRNA/change in abundance in total mRNA (RR = polyRNA/totRNA). Therefore, for each mRNA expressed, the RR value reveals the change in its association with polysomes independently to any change in its total cytoplasmic amount.

Among the 17416 expressed mRNAs, 1151 displayed modified translatability (RR ≥1.50 or RR ≤0.67). The translational changes of one third of mRNA species (148 negatively and 326 positively) were explained by their differential association with polysomes coupled with no significant changes in their total cytoplasmic abundance. A further third of these mRNAs (131 negatively and 266 positively) were consistently present within the polysomal fraction while their total cytoplasmic amount varied. Finally, 194 mRNAs displayed changes in abundance in polysomes together with opposite changes in total mRNA, suggesting that their translation was affected through highly controlled recruitment to polysomes (Figure 2B).

In addition, 66 expressed mRNAs displayed concomitant changes in their abundance both in polysomes and in the cytoplasm, resulting in an unmodified translatability (RR ≥0.67 and RR ≤1.50). This suggests that their translational regulation was not affected by BRCA1.

### Characteristics of Genes Exhibiting Altered Translational Efficiencies on BRCA1 Depletion

Distinct categories of mRNAs that displayed either increased or decreased translatability (RR ≥1.50 or RR ≤0.67) in BRCA1-depleted cells were identified. A gene ontology analysis conducted with Ingenuity Pathway analysis (IPA) software was used to determine whether translationally deregulated genes are significantly enriched in particular functions (Figure 2C).

Interestingly, the most enriched function is cellular maintenance, the function considered today as the major role of BRCA1 contributing to its tumour suppressor activity. We also noticed that the second and third enriched functions, namely embryonic development and lipid metabolism, are two cellular processes previously linked to BRCA1 [21] [22]. In addition, gene ontology analysis revealed that BRCA1 affects translation of genes involved in less expected functions such as cellular movement, nucleic acid metabolism or protein trafficking. To our knowledge, these mechanisms have not to date been clearly associated with BRCA1 tumour suppressor activity and would be worth examining further.

Gene ontology analysis showed that several functions contributing to cell surveillance such as cell cycle arrest, cell death, cellular growth and proliferation, DNA repair and gene expression are enriched in genes translationally controlled by BRCA1. We further explored the expression of several genes reminiscent of these 5 main functions depicted for BRCA1 (Table 1). Analysis of RR values showed that 23 mRNAs were translationally increased (RR ≥1.5) while 12 mRNAs were translationally decreased (RR ≤0.67) in BRCA1-depleted MCF7 cells. Our findings also indicate that the translational changes of the majority of these mRNAs (22 out of 35) in BRCA1-depleted cells are explained by their differential association to polysomes with no significant changes in their total mRNA abundance. In addition, some translationally enhanced mRNAs such as ASH1L, REV1, TRIB3 or CDK6, showed a decrease in total quantity of mRNA, however the fraction of mRNA present on polysomes remained constant. Other mRNAs, such as CBX5, or DDX17, although increased in total abundance, did not efficiently associate with polysomes and were therefore translationally repressed.

**Table 1:** Selected candidate mRNAs that displayed a significantly altered translation in BRCA1-depleted MCF7 cells compared to control MCF7 cells.

Gene Symbol	Gene Title	Poly RNA	Tot RNA	poly/tot	GenBank
	**Gene Expression**				
ACVR1B	activin A receptor, type IB	0,45	0,9	0,5	NM_004302
AKAP5	A kinase (PRKA) anchor protein 5	1,3	0,8	1,7	NM_004857
ASH1L	ash1 (absent, small, or homeotic)-like (Drosophila)	1,1	0,6	1,8	NM_018489
BRWD1	bromodomain and WD repeat domain containing 1	1,7	0,8	2,2	NM_033656
CBX5	chromobox homolog 5 (HP1 alpha homolog, Drosophila)	0,9	2,0	0,5	NM_001127322
CRTC1	CREB regulated transcription coactivator 1	0,9	1,4	0,6	NM_015321
DDX17	DEAD (Asp-Glu-Ala-Asp) box polypeptide 17	0,6	1,9	0,3	NM_006386
HDAC8	histone deacetylase 8	1,5	0,8	1,8	NM_018486
MLL5	myeloid/lymphoid or mixed-lineage leukemia 5	1,7	0,7	2,4	NM_182931
NFE2L3	nuclear factor (erythroid-derived 2)-like 3	1,8	0,8	2,1	NM_004289
PHRF1	PHD and ring finger domains 1	0,5	1,2	0,4	NM_020901
POLR3G	polymerase (RNA) III (DNA directed) polypeptide G (32 kD)	2,0	1,0	2,1	NM_006467
PRKACB	protein kinase, cAMP-dependent, catalytic, beta	0,5	1,4	0,4	NM_207578
THRA	thyroid hormone receptor, alpha	2,2	1,1	1,9	NM_199334
	**Genome Integrity and DNA Repair**				
POLK	polymerase (DNA directed) kappa	2,2	0,9	2,3	NM_016218
REV1	REV1 homolog (S. cerevisiae)	1,0	0,2	4,5	NM_016316
SMC5	structural maintenance of chromosomes 5	2,0	1,0	1,9	NM_015110
SMC6	structural maintenance of chromosomes 6	1,3	0,8	1,7	NM_001142286
TOP1	topoisomerase (DNA) I	1,3	0,7	1,9	NM_003286
	**Cell Death and Survival**				
BHLHE41	basic helix-loop-helix family, member e41	2,6	0,8	3,2	NM_030762
CD47	CD47 molecule	0,9	0,5	1,7	NM_001777
DNASE1L3	deoxyribonuclease I-like 3	1,8	1,0	1,8	NM_004944
FOXO3	forkhead box O3	0,5	1,0	0,5	NM_001455
HIPK2	homeodomain interacting protein kinase 2	1,5	0,9	1,8	NM_022740
TRIB3	tribbles homolog 3 (Drosophila)	1,2	0,5	2,4	NM_021158
	**Cell Growth and Proliferation**				
FAM110B	family with sequence similarity 110, member B	0,4	0,8	0,5	NM_147189
PTK7	protein tyrosine kinase 7	2,1	0,6	3,6	NM_002821
RUNX1	runt-related transcription factor 1	0,9	1,4	0,6	NM_001122607
TRAF4	TNF receptor-associated factor 4	1,0	1,6	0,6	NM_004295
	**Cell Cycle Control**				
CDK6	cyclin-dependent kinase 6	0,9	0,3	2,7	NM_001259
CRY2	cryptochrome 2 (photolyase-like)	1,5	0,9	1,6	NM_021117
GSG2	germ cell associated 2 (haspin)	1,0	1,6	0,6	NM_031965
NDE1	nudE nuclear distribution gene E homolog 1 (A. nidulans)	0,9	1,4	0,6	NM_001143979
RECQL	RecQ protein-like (DNA helicase Q1-like)	1,4	0,8	1,8	NM_002907
TPR	translocated promoter region (to activated MET oncogene)	1,8	0,7	2,5	NM_003292

Genes are clustered into functional groups. Each gene is annotated in the table with the gene symbol, the gene name, the fold change in abundance in polysomal RNA (polyRNA), the fold change in mRNA abundance (totRNA), the relative translatability (poly/tot) and the GenBank accession number.

In addition, this analysis indicates that the mRNAs implicated in genome integrity and DNA repair were all translationally stimulated in BRCA1-depleted MCF7 cells, suggesting a compensatory response to the loss of BRCA1 DNA repair function as recently mentioned [23]. Most of the mRNAs implicated in cell death and survival were also translationally increased, suggesting a similar mechanism of compensation. This is in line with previous reports proposing that loss of functional homology-directed DNA repair through knock-out of BRCA1 might be partially compensated for by other DNA repair mechanisms [24].

### Validation of Gene Expression Alteration by Quantitative RT-PCR and Western Blot

We used RT-qPCR to verify the translational regulation of five candidate genes identified by our microarray analysis presented in Table 1. To this end, we used a total of four independent experiments including one used for the microarray.

**Figure 3:**
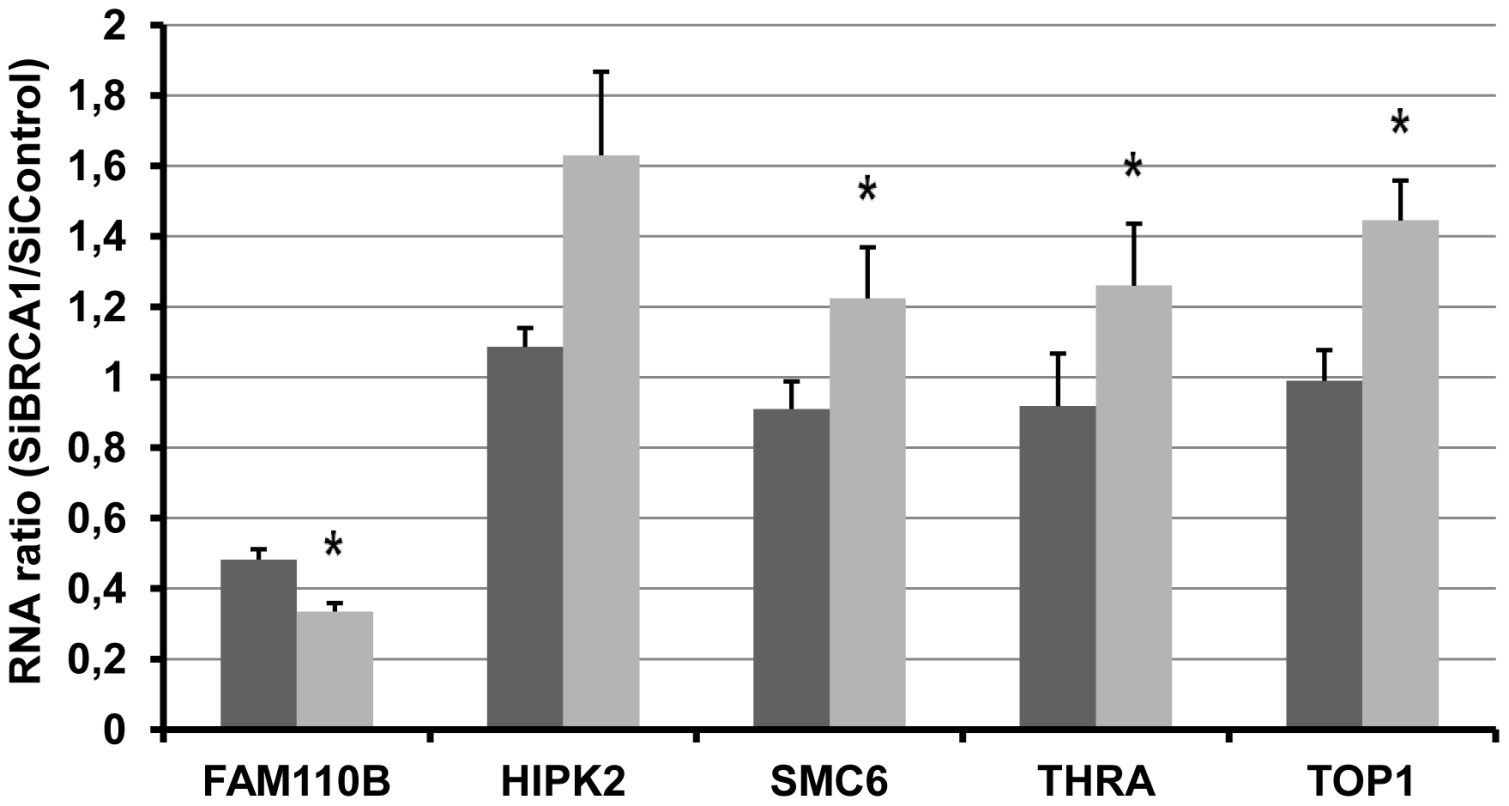
RT-qPCR analyses of differentially translated mRNAs upon BRCA1 depletion. Total RNA and polysomal-associated RNA from MCF7 cells transfected with BRCA1-targetting siRNA or control siRNA were reverse transcribed and five transcripts identified in the microarray analysis were quantified by real time PCR. qPCR analysis was performed in triplicate. Analysis of mRNA levels for each target was normalized to HPRT1 mRNA. For each gene, the polyRNA (grey) and the totalRNA (black) Ratios were determined using the ΔΔCt calculation method. Results are representative of the average RNA ratio ± SEM from four independent experiments. *, *p*<0.05 compared with SiControl.

Upon microarray analysis, four candidates, HIPK2, SMC6, THRA and TOP1, demonstrated a translational induction with a RR ≥1.5 in the absence of changes in total mRNAs. Using RT-qPCR, we showed that polysomal mRNA abundance was increased upon BRCA1 depletion, without a large change in total mRNA level. We therefore confirmed the translational induction of these mRNAs with a clear significance for SMC6, THRA and TOP1 (*p* = 0.05) and a near limit significance for HIPK2 (*p* = 0 07) (Figure 3).

Upon microarray analysis, one candidate, FAM110B, demonstrated a decreased translation with a RR ≤0.67. Results of RT-qPCR confirmed the negative translational regulation of this mRNA.

Previous reports described HIPK2 as a tumour suppressor responsive to DNA damage. Its activation downstream of the ATM and/or ATR kinases leads to cell death and growth suppression [25] [26]. Therefore, HIPK2 shares with BRCA1 some common features. This led us to further investigate the regulation of HIPK2 translation.

**Figure 4:**
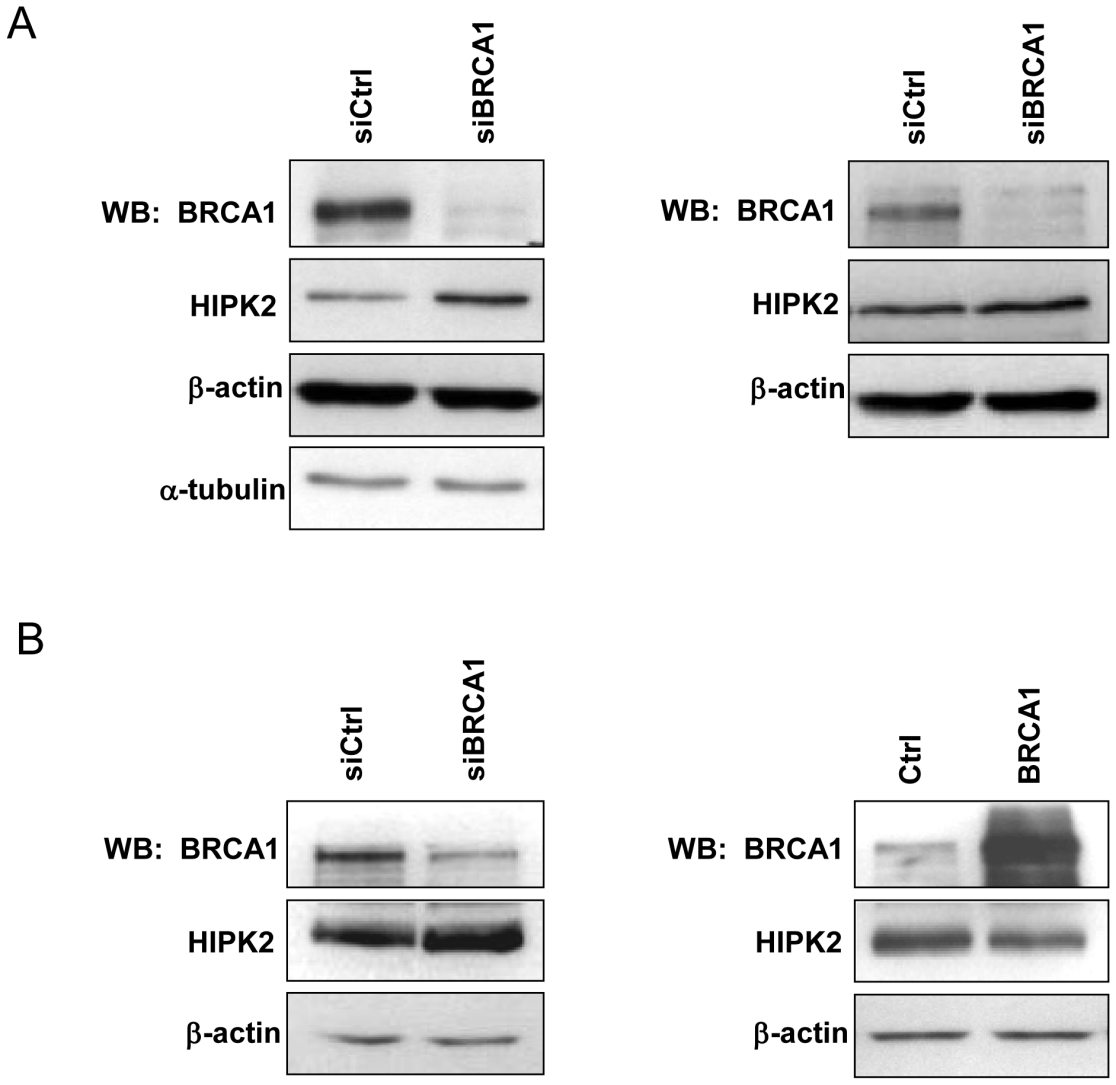
Analyses of HIPK2 protein predicted to be modified by BRCA1 depletion. A/MCF7 cells transfected with BRCA1-targetting siRNA or control siRNA and total protein extracts were collected 72 h later and subjected to immunoblot analysis for HIPK2 protein, using antibodies provided by Epitomics (Left) and Aviva (Right). B/293T cells transfected with BRCA1-targetting siRNA or control siRNA (Left) or transfected with BRCA1 expressing plasmid or empty plasmid as control (Right) were subjected to immunoblot analysis for HIPK2 protein, using antibodies provided by Epitomics. Blots used β-actin and/or α-tubulin as loading controls. The blots shown are representative of at least three independent experiments.

To determine whether the BRCA1-dependent change in translational activity of HIPK2 mRNA corresponds with a change in its protein product, immunoblots were performed for BRCA1-depleted and control MCF7 cells. As shown in Figure 4A, the HIPK2 protein is induced in BRCA1-depleted MCF7 cells, while β-actin and α-tubulin are unaffected.

To further ascertain the role of BRCA1 on HIPK2 protein abundance, a second anti-HIPK2 antibody was used to detect HIPK2 protein in BRCA1-depleted and control MCF7 cells. Results confirmed increased HIPK2 protein abundance under BRCA1 depletion (Figure 4A). We also analyzed the impact of RNAi-mediated gene silencing of BRCA1 in non-mammary cells, namely 293T cells. Similar to what has been observed in MCF7 cells, HIPK2 protein is induced in BRCA1-depleted 293T cells. Accordingly, 293T cells that over-expressed BRCA1 exhibited a decreased quantity of HIPK2 protein (Figure 4B).

## Discussion

Here, we have used a microarray analysis of polysome-associated mRNA from BRCA1-depleted MCF7 cells to provide a genome-wide overview of the role played by BRCA1 in translational regulation. We calculated the relative translatability through the RR ratio (RR = polyRNA/totRNA) to reveal, for each mRNA, the change in its association with polysomes independently to any change in its total cytoplasmic amount. We demonstrate that BRCA1 modifies the translational regulation of about 7% of genes expressed in MCF7 cells. Our findings also indicate that the translational changes of many mRNAs in BRCA1-depleted cells are explained by their differential association with polysomes coupled with no significant changes in their total mRNA abundance. The modified translatability of other mRNA species originated from modified total mRNA abundance counteracted by consistent presence on polysomes. Interestingly, some mRNAs displayed changes in abundance in polysomes together with opposite changes in total mRNA, suggesting that their recruitment to polysomes is highly controlled by BRCA1.

Gene ontology analysis revealed that the most enriched function of the genes that are translationally regulated is cellular maintenance, which is considered today as the major role of BRCA1 contributing to its tumour suppressor activity [6]. Accordingly, gene ontology analysis showed that several cellular processes participating in cellular maintenance, such as cell cycle arrest, cell death, cellular growth and proliferation, DNA repair and gene expression, are enriched in genes translationally controlled by BRCA1. We used an RT-qPCR approach to ascertain the significant changes in mRNA’s polysome association of 5 genes identified through our microarray and selected for their participation in main functions of BRCA1 implicated in cell surveillance. In addition, some translationally deregulated genes are involved in lipid metabolism and should be further examined as BRCA1 has been recently implicated in negative control of lipid metabolism [20] [27]. Finally, this study revealed that BRCA1 impacts translation of genes involved in several cellular functions that are not currently clearly linked to BRCA1, such as cellular movement, nucleic acid metabolism or protein trafficking. The identity of these latter genes may be indicative of novel functions for BRCA1.

To our knowledge, genome-wide translational control of mRNAs by BRCA1 has not been described previously. Studies performing gene expression microarray analysis with RNAi-depleted BRCA1 MCF7, T47D or HeLa cells identified a number of genes regulated by BRCA1 at the transcriptional level [28] [29] [30]. Comparing these genes with those found translationally deregulated in our study, few, if any genes, were found to be affected through both translational and transcriptional mechanisms in absence of BRCA1. This suggests that BRCA1 differentially regulates transcription and translation of cellular genes. The difference we observe between changes in transcription and translation is consistent with previous reports involving eukaryotic cells [31]. By comparing protein expression levels with RNA levels, a recent proteomic study using mouse Brca1-deficient mammary tumours clearly showed that RNA levels can be discordant with their protein expression [23]. These results underscore the importance of studying not only transcriptional regulation but also translational regulation to understand BRCA1-dependent biological processes.

Large-scale profiling of mRNA translation efficiencies during mitotic cycle or cell stress such as hypoxia, apoptosis, endoplasmic reticulum stress or UVB DNA damage, has revealed that certain mRNAs evade global inhibition of protein synthesis [32] [33] [34] [35] [36]. Furthermore, the 5′UTR of these mRNAs plays a crucial role in the mechanism of selective mRNA translation. Many of these mRNAs are translated using alternative mechanisms of translation initiation, such as the IRES (Internal Ribosome Entry Site)-dependent mechanism [37] [38]. The translation of these mRNAs is maintained or increased during stress through activity of trans-acting factors that act either as RNA chaperones or as adaptor proteins [39]. Several of these trans-acting factors, called ITAF (IRES-Trans acting factor) when implicated in IRES-dependent translation, are regulated by their sub-cellular localization [40]. Therefore, we can hypothesize that BRCA1 plays a role of trans-acting factor with translational activity that may vary greatly depending on the physiological status of the cell, e.g. upon induction of stress response or DNA damage. Thus, like other trans-acting factors such as HuR, PTB, hnRNP A1 and RBM4 [37] [41] [42], BRCA1 may regulate translation of different subsets of mRNA depending on its sub-cellular localization that is modified in response to cell stress. Given its role as a tumour suppressor and its stimulated nucleo-cytoplasmic shuttling properties after DNA damage [9], it is possible that in response to DNA damaging agents, BRCA1 preferentially regulates the translation of genes involved in DNA repair. Furthermore, we may propose the hypothesis that other genes not observed as regulated by BRCA1 in this study may be deregulated due to stress conditions specific to these genes or pathways and their interaction with BRCA1. Since translation is the last step in the flow of expression of genetic information, BRCA1-dependent regulation at the translational level would allow for an immediate and rapid response to changes in physiological conditions such as DNA damage without the involvement of mRNA synthesis and transport.

We previously showed that BRCA1 regulates global translation in a PABP1-dependent manner. We demonstrate here that BRCA1 regulates the translation of a limited number of mRNAs. Because PABP1 acts as a general translational factor, we asked how BRCA1 selectively impacts the translation of only some mRNAs species. We first examined whether the proteins encoded by these mRNAs present some common features. Using IPA software, we found that these proteins participate in many cellular pathways, and are not restricted to any particular gene ontology function such as transcription regulators or kinases. It is possible that there are regulatory *cis*-elements in these mRNAs, such as a defined sequence in the 5′ or 3′untranslated region (UTR). We here compared the up- and down-regulated mRNA sequences in BRCA1-depleted MCF7 cells to investigate the possibility of some common features of the 5′UTRs. The analyses of 5′UTR sequences retrieved from UTRdb [43] for the positively, negatively and not (neutral) regulated mRNAs indicated that their GC content distributions differ for the 3 groups (Figure S1; Tables S1 and S2) while the length distributions are similar as assessed by a two-sample Kolmogorov-Smirnov test (Figure S2; Tables S3 and S4). These data suggest that the 5′UTR of mRNAs which are translationally regulated by BRCA1 share common features. It would be worth examining whether these 5′UTRs contain common mRNA motifs that could be recognized by BRCA1 interacting partners and/or BRCA1 itself. This hypothesis will be examined in future work.

Here we show that BRCA1 can act both as a translational repressor and as a translational activator. Among the genes whose translational level is significantly changed in response to BRCA1 depletion in MCF7 cells, we have selected those that were shown to play a role in cancer and we discuss below results concerning 5 of them.

HIPK2 is an emerging regulator of cell growth and apoptosis in various cell types and tissues, but regulation of HIPK2 remains largely obscure [44] [26]. HIPK2 has been described as a potential tumour suppressor and DNA damage-responsive kinase [45]. However, recent reports also indicate that HIPK2 can stimulate cell growth and that its expression level correlates with tumour progression. For example, in pilocytic astrocytoma, the HIPK2 gene is frequently amplified and HIPK2 over-expression stimulates cell growth [46]. Moreover, strong HIPK2 immunostaining in cervical cancer tissues was hypothesized to correlate with tumour progression [47]. In this context, our findings that BRCA1 negatively regulates the HIPK2 protein abundance suggest that BRCA1 modulates preferentially the growth-driving functions of HIPK2.

The widely expressed nuclear protein SMC6 (Structural Maintenance of Chromosome 6) forms the SMC5/SMC6 complex with SMC5 and is involved in DNA double-strand break repair [48]. Interestingly, this complex has been shown to facilitate telomere homologous recombination and elongation in cancer cells, indicating that the SMC5/6 complex is required for telomere maintenance [49]. We here propose that through negative translational regulation of SMC6, BRCA1 may protect cells from telomere elongation that is a mark of proliferative status. The fact that SMC5 was also negatively regulated by BRCA1 reinforces this model.

TOP1 (DNA Topoisomerase 1) controls and alters the topologic states of DNA during transcription. It is specifically inhibited by camptothecin (CPT), a drug that participates in current therapeutic protocols and particularly to breast tumour therapy, and by topotecan, a drug that is under clinical trial [50]. Several studies have proposed links between TOP1 and BRCA1. Markedly, BRCA1 has been shown to participate to the transcription-induced degradation of TOP1 [51]. Here, we also show that BRCA1 negatively regulates TOP1 suggesting that the control of TOP1 translation plays a part in the global down-regulation of TOP1 by BRCA1. Another critical point concerns the resistance to camptothecin (CPT) of some BRCA1-deficient breast tumours. Despite early reports that suggested the potential value of determining TOP1 levels in tumours, current therapeutic protocols do not take tumour expression levels of TOP1 protein or mRNA into account before giving CPT-based therapies [52]. In this critical context, it could be of great interest to examine the expression of BRCA1 that may function as a potential regulator of TOP1 expression and therefore be determinant for the efficiency of the anti-tumour treatment. A proteomic study using mouse Brca1-deficient mammary tumours has recently reported TOP1 as up-regulated [23] therefore strengthening our findings.

Historically, THRA (Thyroid Hormone Receptor Alpha) has been used to delineate the locus linked to breast cancer including *BRCA1*. A detailed deletion mapping of chromosome segment 17q12–21 in sporadic breast tumours showed that the pattern of LOH covered the BRCA1 and THRA locus [53]. Since then, several genetic analyses have shown some interplay between BRCA1 and THRA genes in breast cancers. Today, data from genetic studies suggest a new functional link between these two genes, and highlight the crucial impact of the expression regulation on cancer progression [54]. From a functional point of view, THRA encodes 3,30,5-triiodo-L-thyronine-binding thyroid hormone receptor isoform a1 (TRa1) that is predominantly expressed in the brain and adipose tissue. TRa1 plays a significant role in adipogenesis and maintenance of mature adipocyte functions. The use of TRa1 knock-in mutant mice has clearly shown that TRa1 plays a critical role in regulation of lipid homeostasis in white adipose tissue [55]. Gene expression profiling revealed a strong induction of genes involved in lipolysis, lipogenesis, and glucose handling as, for example, induction of the expression of acetyl CoA-carboxylase alpha (ACCA) [55]. We propose here a new model in which negative control of lipid synthesis by BRCA1 would be mediated not only through the BRCA1-ACCA interaction as we previously described [20] [56] [57], but also *via* the BRCA1-dependent translational regulation of THRA.

Progression between the different cell cycle phases is controlled at specific checkpoints. Centrosomes and microtubules (MTs) are critical for the propagation of a stable genome through cell division. Several human pathologies, most notably cancer, have been related to centrosome abnormalities and MT defects. FAM110 (FAMily with sequence similarity 110) proteins have recently been identified as interacting with CSPP (centrosome/spindle pole-associated protein) using yeast two-hybrid assays. In the interphase, FAM110B showed both nuclear and cytoplasmic localization, accumulating at the centrosome/MTOC (MT organizing center). In mitosis, FAM110B slightly accumulates at the centrosomes and spindle poles [58]. Over-expression of FAM110B in HEK293T cells leads to an increase of diploid/G1 cells and impairs cell cycle progression through G1. The mechanisms causing the arrest are not known. The positive effect of BRCA1 on FAM110B translation that we uncovered in this study suggests that a functional interplay between BRCA1 which is largely implicated in cell cycle checkpoints and FAM110B, may contribute to a strict cell cycle control.

The BRCA1 protein is inactivated in most familial cases with *BRCA1* mutations and is under-expressed in about 30% of sporadic breast cancers, mainly in high grade tumours and in “basal-like” or “triple-negative” (ER-, PR-, HER2-) tumours. Because the BRCA1-depleted MCF7 cells used in this study mimic the physiological status of BRCA1 in breast cancers, we asked whether some translationally deregulated mRNAs encode potential therapeutic targets. Using IPA software, we found a number of drug targets to be regulated in the BRCA1-depleted cells (Table S5). They include the up-regulated HDAC8, THRA, TOP1 and CDK6 that participate to the 5 main functions depicted for BRCA1. HDAC8 is involved in chromatin remodelling by histone deacetylation. Inhibition of HDAC8 results in α-tubulin acetylation and therefore sensitizes breast cancer cells to tubulin-polymerizing agents [59]. Several kinases are up-regulated in the absence of BRCA1, including CDK6 and PIK3CB. CDK4/6 inhibitors that interrupt cell cycle have therapeutic efficacy in combination with endocrine-based therapy in patients with steroid hormone receptor positive breast cancer [60]. The PIK3CB kinase belongs to the well-investigated PI3K pro-proliferative pathway and is a target of several inhibitory drugs currently under trial [61]. Additional genes identified here are implicated in functions unrelated to BRCA1 and may constitute some new avenues to explore, leading to new therapeutic targets for BRCA1-deficient cancers.

It is well recognized that abnormal translation is a fundamental characteristic of tumour cells and a potential target for cancer treatment [62] [63]. In addition, therapeutic implications of BRCA1 dysfunction are at this time largely theoretical and today it is crucial to uncover novel efficient therapeutics. In this context, BRCA1-dependent translational regulation represents a novel way by which BRCA1 exerts its global role in cell surveillance, and the identification of BRCA1’s translational targets should lead to the discovery of new markers of tumorigenesis and new therapeutic targets.

## Supporting Information

Figure S1 **Distribution of GC content for the 3 sets of 5′UTRs.** The x-axis lists the 11 classes of GC content (in %). The y-axis shows the amount of UTRs belonging to the class (in %). The sets are indicated in black (positive), white (negative) and grey (neutral).
(DOC)

Figure S2 **Distribution of length for the 3 sets of 5′UTRs.** The x-axis lists the 21 classes of length (in nucleotides). The y-axis shows the amount of UTRs belonging to the class (in %). The sets are indicated in black (positive), white (negative) and grey (neutral).
(DOC)

Table S1 **Two-sample Kolmogorov–Smirnov test on GC content for the 3 sets of 5′UTRs**
(DOC)

Table S2 **Statistical parameters on GC content for the 3 sets of 5′UTRs**
(DOC)

Table S3 **Two-sample Kolmogorov–Smirnov test on length for the 3 sets of 5′UTRs**
(DOC)

Table S4 **Statistical parameters on length for the 3 sets of 5′UTRs**
(DOC)

Table S5 **IPA drug targets that displayed an altered translation in BRCA1-depleted MCF7 cells compared to control MCF7 cells.** Each gene is annotated in the table with the gene symbol, the relative translatability (polyRNA/totRNA), the GenBank accession number, the gene name, the family and the IPA drugs.
(XLS)

## References

[1] Stratton MR, Rahman N (2008) The emerging landscape of breast cancer susceptibility. Nat Genet 40: 17–22. 18163131 10.1038/ng.2007.53

[2] Futreal PA, Liu Q, Shattuck-Eidens D, Cochran C, Harshman K, et al (1994) BRCA1 mutations in primary breast and ovarian carcinomas. Science 266: 120–122. 7939630 10.1126/science.7939630

[3] Rio PG, Maurizis JC, Peffault de Latour M, Bignon YJ, Bernard-Gallon DJ (1999) Quantification of BRCA1 protein in sporadic breast carcinoma with or without loss of heterozygosity of the BRCA1 gene. Int J Cancer 80: 823–826. 10074913 10.1002/(sici)1097-0215(19990315)80:6<823::aid-ijc5>3.0.co;2-3

[4] Rakha EA, El-Sheikh SE, Kandil MA, El-Sayed ME, Green AR, et al (2008) Expression of BRCA1 protein in breast cancer and its prognostic significance. Hum Pathol 39: 857–865. 18400253 10.1016/j.humpath.2007.10.011

[5] Wilson CA, Ramos L, Villasenor MR, Anders KH, Press MF, et al (1999) Localization of human BRCA1 and its loss in high-grade, non-inherited breast carcinomas. Nat Genet 21: 236–240. 9988281 10.1038/6029

[6] Venkitaraman AR (2002) Cancer susceptibility and the functions of BRCA1 and BRCA2. Cell 108: 171–182. 11832208 10.1016/s0092-8674(02)00615-3

[7] Fabbro M, Henderson BR (2003) Regulation of tumor suppressors by nuclear-cytoplasmic shuttling. Exp Cell Res 282: 59–69. 12531692 10.1016/s0014-4827(02)00019-8

[8] Wang H, Yang ES, Jiang J, Nowsheen S, Xia F (2010) DNA damage-induced cytotoxicity is dissociated from BRCA1’s DNA repair function but is dependent on its cytosolic accumulation. Cancer Res 70: 6258–6267. 20631074 10.1158/0008-5472.CAN-09-4713 PMC2912952

[9] Okada S, Ouchi T (2003) Cell cycle differences in DNA damage-induced BRCA1 phosphorylation affect its subcellular localization. J Biol Chem 278: 2015–2020. 12427729 10.1074/jbc.M208685200

[10] Fabbro M, Schuechner S, Au WW, Henderson BR (2004) BARD1 regulates BRCA1 apoptotic function by a mechanism involving nuclear retention. Exp Cell Res 298: 661–673. 15265711 10.1016/j.yexcr.2004.05.004

[11] Yang ES, Xia F (2010) BRCA1 16 years later: DNA damage-induced BRCA1 shuttling. FEBS J 277: 3079–3085. 20608971 10.1111/j.1742-4658.2010.07734.x PMC6158790

[12] Dizin E, Ray H, Suau F, Voeltzel T, Dalla Venezia N (2008) Caspase-dependent BRCA1 cleavage facilitates chemotherapy-induced apoptosis. Apoptosis 13: 237–246. 18071904 10.1007/s10495-007-0167-4

[13] Laulier C, Barascu A, Guirouilh-Barbat J, Pennarun G, Le Chalony C, et al (2011) Bcl-2 inhibits nuclear homologous recombination by localizing BRCA1 to the endomembranes. Cancer Res 71: 3590–3602. 21444675 10.1158/0008-5472.CAN-10-3119

[14] Dizin E, Gressier C, Magnard C, Ray H, Decimo D, et al (2006) BRCA1 interacts with poly(A)-binding protein: implication of BRCA1 in translation regulation. J Biol Chem 281: 24236–24246. 16782705 10.1074/jbc.M602176200

[15] Scully R, Chen J, Plug A, Xiao Y, Weaver D, et al (1997) Association of BRCA1 with Rad51 in mitotic and meiotic cells. Cell 88: 265–275. 9008167 10.1016/s0092-8674(00)81847-4

[16] Belin S, Hacot S, Daudignon L, Therizols G, Pourpe S, et al.. (2010) Purification of ribosomes from human cell lines. Curr Protoc Cell Biol Chapter 3: Unit 3 40. 10.1002/0471143030.cb0340s49 21154551

[17] Nguyen-Lefebvre AT, Gonin-Giraud S, Scherl A, Arboit P, Granger L, et al (2011) Identification of human, rat and chicken ribosomal proteins by a combination of two-dimensional polyacrylamide gel electrophoresis and mass spectrometry. J Proteomics 74: 167–185. 21055487 10.1016/j.jprot.2010.10.007

[18] Rivera CI, Lloyd RE (2008) Modulation of enteroviral proteinase cleavage of poly(A)-binding protein (PABP) by conformation and PABP-associated factors. Virology 375: 59–72. 18321554 10.1016/j.virol.2008.02.002 PMC2409284

[19] Nousch M, Reed V, Bryson-Richardson RJ, Currie PD, Preiss T (2007) The eIF4G-homolog p97 can activate translation independent of caspase cleavage. RNA 13: 374–384. 17237356 10.1261/rna.372307 PMC1800516

[20] Moreau K, Dizin E, Ray H, Luquain C, Lefai E, et al (2006) BRCA1 affects lipid synthesis through its interaction with acetyl-CoA carboxylase. J Biol Chem 281: 3172–3181. 16326698 10.1074/jbc.M504652200

[21] Brunet J, Vazquez-Martin A, Colomer R, Grana-Suarez B, Martin-Castillo B, et al (2008) BRCA1 and acetyl-CoA carboxylase: the metabolic syndrome of breast cancer. Mol Carcinog 47: 157–163. 17620310 10.1002/mc.20364

[22] Hakem R, de la Pompa JL, Mak TW (1998) Developmental studies of Brca1 and Brca2 knock-out mice. J Mammary Gland Biol Neoplasia 3: 431–445. 10819537 10.1023/a:1018792200700

[23] Warmoes M, Jaspers JE, Pham TV, Piersma SR, Oudgenoeg G, et al.. (2012) Proteomics of mouse BRCA1-deficient mammary tumors identifies DNA repair proteins with potential diagnostic and prognostic value in human breast cancer. Mol Cell Proteomics 11: M111 013334. 10.1074/mcp.M111.013334 PMC3394939 22366898

[24] Helleday T, Petermann E, Lundin C, Hodgson B, Sharma RA (2008) DNA repair pathways as targets for cancer therapy. Nat Rev Cancer 8: 193–204. 18256616 10.1038/nrc2342

[25] Hofmann TG, Glas C, Bitomsky N (2013) HIPK2: A tumour suppressor that controls DNA damage-induced cell fate and cytokinesis. Bioessays 35: 55–64. 23169233 10.1002/bies.201200060

[26] D’Orazi G, Rinaldo C, Soddu S (2012) Updates on HIPK2: a resourceful oncosuppressor for clearing cancer. J Exp Clin Cancer Res 31: 63. 22889244 10.1186/1756-9966-31-63 PMC3432601

[27] Ortega FJ, Moreno-Navarrete JM, Mayas D, Garcia-Santos E, Gomez-Serrano M, et al (2012) Breast cancer 1 (BrCa1) may be behind decreased lipogenesis in adipose tissue from obese subjects. PLoS One 7: e33233. 22666314 10.1371/journal.pone.0033233 PMC3364252

[28] Gorski JJ, Savage KI, Mulligan JM, McDade SS, Blayney JK, et al (2011) Profiling of the BRCA1 transcriptome through microarray and ChIP-chip analysis. Nucleic Acids Res 39: 9536–9548. 21880590 10.1093/nar/gkr679 PMC3239190

[29] Gorski JJ, James CR, Quinn JE, Stewart GE, Staunton KC, et al (2010) BRCA1 transcriptionally regulates genes associated with the basal-like phenotype in breast cancer. Breast Cancer Res Treat 122: 721–731. 19882246 10.1007/s10549-009-0565-0

[30] Lamber EP, Horwitz AA, Parvin JD (2010) BRCA1 represses amphiregulin gene expression. Cancer Res 70: 996–1005. 20103632 10.1158/0008-5472.CAN-09-2842 PMC2816672

[31] Ideker T, Thorsson V, Ranish JA, Christmas R, Buhler J, et al (2001) Integrated genomic and proteomic analyses of a systematically perturbed metabolic network. Science 292: 929–934. 11340206 10.1126/science.292.5518.929

[32] Qin X, Sarnow P (2004) Preferential translation of internal ribosome entry site-containing mRNAs during the mitotic cycle in mammalian cells. J Biol Chem 279: 13721–13728. 14739278 10.1074/jbc.M312854200

[33] Thomas JD, Johannes GJ (2007) Identification of mRNAs that continue to associate with polysomes during hypoxia. RNA 13: 1116–1131. 17488873 10.1261/rna.534807 PMC1894931

[34] Bushell M, Stoneley M, Kong YW, Hamilton TL, Spriggs KA, et al (2006) Polypyrimidine tract binding protein regulates IRES-mediated gene expression during apoptosis. Mol Cell 23: 401–412. 16885029 10.1016/j.molcel.2006.06.012

[35] Powley IR, Kondrashov A, Young LA, Dobbyn HC, Hill K, et al (2009) Translational reprogramming following UVB irradiation is mediated by DNA-PKcs and allows selective recruitment to the polysomes of mRNAs encoding DNA repair enzymes. Genes Dev 23: 1207–1220. 19451221 10.1101/gad.516509 PMC2685536

[36] Kawai T, Fan J, Mazan-Mamczarz K, Gorospe M (2004) Global mRNA stabilization preferentially linked to translational repression during the endoplasmic reticulum stress response. Mol Cell Biol 24: 6773–6787. 15254244 10.1128/MCB.24.15.6773-6787.2004 PMC444849

[37] Spriggs KA, Stoneley M, Bushell M, Willis AE (2008) Re-programming of translation following cell stress allows IRES-mediated translation to predominate. Biol Cell 100: 27–38. 18072942 10.1042/BC20070098

[38] Komar AA, Hatzoglou M (2011) Cellular IRES-mediated translation: the war of ITAFs in pathophysiological states. Cell Cycle 10: 229–240. 21220943 10.4161/cc.10.2.14472 PMC3048795

[39] King HA, Cobbold LC, Willis AE (2010) The role of IRES trans-acting factors in regulating translation initiation. Biochem Soc Trans 38: 1581–1586. 21118130 10.1042/BST0381581

[40] Lewis SM, Holcik M (2008) For IRES trans-acting factors, it is all about location. Oncogene 27: 1033–1035. 17767196 10.1038/sj.onc.1210777

[41] Lewis SM, Veyrier A, Hosszu Ungureanu N, Bonnal S, Vagner S, et al (2007) Subcellular relocalization of a trans-acting factor regulates XIAP IRES-dependent translation. Mol Biol Cell 18: 1302–1311. 17287399 10.1091/mbc.E06-06-0515 PMC1838995

[42] Lin JC, Hsu M, Tarn WY (2007) Cell stress modulates the function of splicing regulatory protein RBM4 in translation control. Proc Natl Acad Sci U S A 104: 2235–2240. 17284590 10.1073/pnas.0611015104 PMC1893002

[43] Pesole G, Liuni S, Grillo G, Licciulli F, Larizza A, et al (2000) UTRdb and UTRsite: specialized databases of sequences and functional elements of 5′ and 3′ untranslated regions of eukaryotic mRNAs. Nucleic Acids Res 28: 193–196. 10592223 10.1093/nar/28.1.193 PMC102415

[44] Sombroek D, Hofmann TG (2009) How cells switch HIPK2 on and off. Cell Death Differ 16: 187–194. 18974774 10.1038/cdd.2008.154

[45] Wei G, Ku S, Ma GK, Saito S, Tang AA, et al (2007) HIPK2 represses beta-catenin-mediated transcription, epidermal stem cell expansion, and skin tumorigenesis. Proc Natl Acad Sci U S A 104: 13040–13045. 17666529 10.1073/pnas.0703213104 PMC1936219

[46] Deshmukh H, Yeh TH, Yu J, Sharma MK, Perry A, et al (2008) High-resolution, dual-platform aCGH analysis reveals frequent HIPK2 amplification and increased expression in pilocytic astrocytomas. Oncogene 27: 4745–4751. 18408760 10.1038/onc.2008.110

[47] Al-Beiti MA, Lu X (2008) Expression of HIPK2 in cervical cancer: correlation with clinicopathology and prognosis. Aust N Z J Obstet Gynaecol 48: 329–336. 18532967 10.1111/j.1479-828X.2008.00874.x

[48] De Piccoli G, Torres-Rosell J, Aragon L (2009) The unnamed complex: what do we know about Smc5-Smc6? Chromosome Res 17: 251–263. 19308705 10.1007/s10577-008-9016-8

[49] Potts PR, Yu H (2007) The SMC5/6 complex maintains telomere length in ALT cancer cells through SUMOylation of telomere-binding proteins. Nat Struct Mol Biol 14: 581–590. 17589526 10.1038/nsmb1259

[50] Lin NU, Eierman W, Greil R, Campone M, Kaufman B, et al (2011) Randomized phase II study of lapatinib plus capecitabine or lapatinib plus topotecan for patients with HER2-positive breast cancer brain metastases. J Neurooncol 105: 613–620. 21706359 10.1007/s11060-011-0629-y

[51] Sordet O, Larochelle S, Nicolas E, Stevens EV, Zhang C, et al (2008) Hyperphosphorylation of RNA polymerase II in response to topoisomerase I cleavage complexes and its association with transcription- and BRCA1-dependent degradation of topoisomerase I. J Mol Biol. 381: 540–549. 10.1016/j.jmb.2008.06.028 PMC2754794 18588899

[52] Pommier Y (2006) Topoisomerase I inhibitors: camptothecins and beyond. Nat Rev Cancer 6: 789–802. 16990856 10.1038/nrc1977

[53] Nagai MA, Yamamoto L, Salaorni S, Pacheco MM, Brentani MM, et al (1994) Detailed deletion mapping of chromosome segment 17q12–21 in sporadic breast tumours. Genes Chromosomes Cancer 11: 58–62. 7529047 10.1002/gcc.2870110109

[54] Lamy PJ, Fina F, Bascoul-Mollevi C, Laberenne AC, Martin PM, et al (2011) Quantification and clinical relevance of gene amplification at chromosome 17q12-q21 in human epidermal growth factor receptor 2-amplified breast cancers. Breast Cancer Res 13: R15. 21288332 10.1186/bcr2824 PMC3109584

[55] Zhu X, Cheng SY (2010) New insights into regulation of lipid metabolism by thyroid hormone. Curr Opin Endocrinol Diabetes Obes 17: 408–413. 20644471 10.1097/MED.0b013e32833d6d46 PMC3457777

[56] Ray H, Moreau K, Dizin E, Callebaut I, Venezia ND (2006) ACCA phosphopeptide recognition by the BRCT repeats of BRCA1. J Mol Biol 359: 973–982. 16698035 10.1016/j.jmb.2006.04.010

[57] Magnard C, Bachelier R, Vincent A, Jaquinod M, Kieffer S, et al (2002) BRCA1 interacts with acetyl-CoA carboxylase through its tandem of BRCT domains. Oncogene 21: 6729–6739. 12360400 10.1038/sj.onc.1205915

[58] Hauge H, Patzke S, Aasheim HC (2007) Characterization of the FAM110 gene family. Genomics 90: 14–27. 17499476 10.1016/j.ygeno.2007.03.002

[59] Ramaswamy B, Fiskus W, Cohen B, Pellegrino C, Hershman DL, et al (2012) Phase I-II study of vorinostat plus paclitaxel and bevacizumab in metastatic breast cancer: evidence for vorinostat-induced tubulin acetylation and Hsp90 inhibition in vivo. Breast Cancer Res Treat 132: 1063–1072. 22200869 10.1007/s10549-011-1928-x PMC3486521

[60] Lange CA, Yee D (2011) Killing the second messenger: targeting loss of cell cycle control in endocrine-resistant breast cancer. Endocr Relat Cancer 18: C19–24. 21613412 10.1530/ERC-11-0112 PMC3924782

[61] Bendell JC, Rodon J, Burris HA, de Jonge M, Verweij J, et al (2012) Phase I, dose-escalation study of BKM120, an oral pan-Class I PI3K inhibitor, in patients with advanced solid tumors. J Clin Oncol 30: 282–290. 22162589 10.1200/JCO.2011.36.1360

[62] Bilanges B, Stokoe D (2007) Mechanisms of translational deregulation in human tumors and therapeutic intervention strategies. Oncogene 26: 5973–5990. 17404576 10.1038/sj.onc.1210431

[63] Silvera D, Formenti SC, Schneider RJ (2010) Translational control in cancer. Nat Rev Cancer 10: 254–266. 20332778 10.1038/nrc2824

