# Supplementary material for: BRCA1-Dependent Translational Regulation in Breast Cancer Cells
Source: PLoS One. 2013 Jun 21;8(6):e67313. doi: 10.1371/journal.pone.0067313 (PMC3689694; doi:10.1371/journal.pone.0067313)

**Figure S1.**

**Distribution of GC content for the 3 sets of 5’UTRs.**

The x-axis lists the 11 classes of GC content (in %). The y-axis shows the amount of UTRs belonging to the class (in %). The sets are indicated in black (positive), white (negative) and grey (neutral).


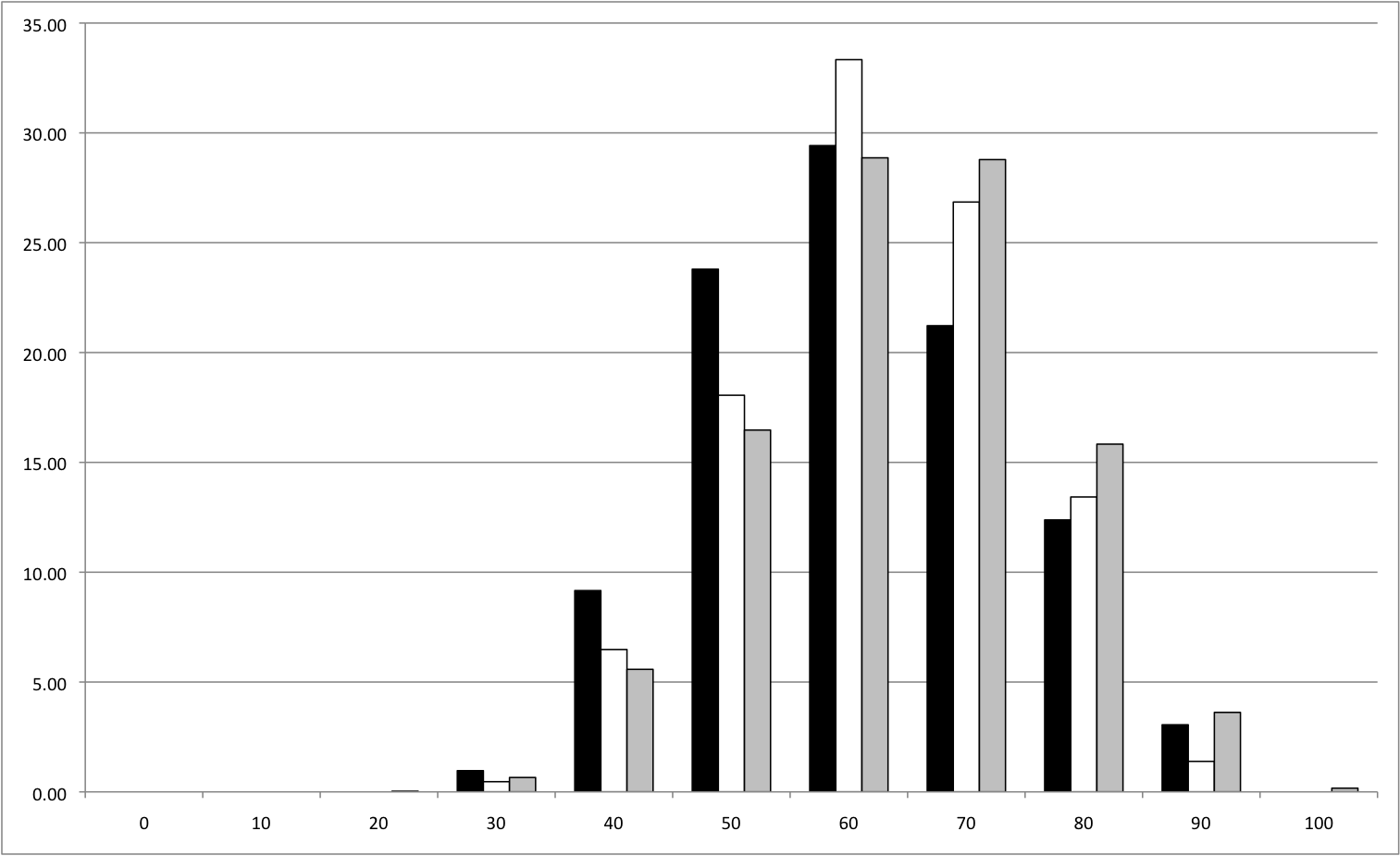

Supplement: Figure S1 — Distribution of GC content for the 3 sets of 5′UTRs. The x-axis lists the 11 classes of GC content (in %). The y-axis shows the amount of UTRs belonging to the class (in %). The sets are indicated in black (positive), white (negative) and grey (neutral). (DOC) [file pone.0067313.s001.doc]
