# Supplementary material for: BRCA1-Dependent Translational Regulation in Breast Cancer Cells
Source: PLoS One. 2013 Jun 21;8(6):e67313. doi: 10.1371/journal.pone.0067313 (PMC3689694; doi:10.1371/journal.pone.0067313)
Supplement: Table S1 — Two-sample Kolmogorov–Smirnov test on GC content for the 3 sets of 5′UTRs (DOC) [file pone.0067313.s003.doc]

**Table S1.**

**Two-sample Kolmogorov–Smirnov test on GC content for the 3 sets of 5’UTRs**

|  | D | P-value | H0 (P>0.05) |
| --- | --- | --- | --- |
| Pos. vs neg. | 0.0951 | 1.99E-02 | reject |
| Pos. vs neu. | 0.1392 | 1.28E-10 | reject |
| Neg. vs neu. | 0.1015 | 3.18E-04 | reject |
