# Supplementary material for: BRCA1-Dependent Translational Regulation in Breast Cancer Cells
Source: PLoS One. 2013 Jun 21;8(6):e67313. doi: 10.1371/journal.pone.0067313 (PMC3689694; doi:10.1371/journal.pone.0067313)
Supplement: Table S2 — Statistical parameters on GC content for the 3 sets of 5′UTRs (DOC) [file pone.0067313.s004.doc]

**Table S2.**

**Statistical parameters on GC content for the 3 sets of 5’UTRs**

|  | Positive | Negative | Neutral |
| --- | --- | --- | --- |
| Mean | 56.06 | 57.55 | 59.26 |
| Median | 55.68 | 58.22 | 59.55 |
| Sandard dev. | 12.45 | 11.22 | 12.08 |
| Min | 26.83 | 25.00 | 12.73 |
| Max | 88.32 | 84.09 | 100.00 |
