# Supplementary material for: BRCA1-Dependent Translational Regulation in Breast Cancer Cells
Source: PLoS One. 2013 Jun 21;8(6):e67313. doi: 10.1371/journal.pone.0067313 (PMC3689694; doi:10.1371/journal.pone.0067313)
Supplement: Table S3 — Two-sample Kolmogorov–Smirnov test on length for the 3 sets of 5′UTRs (DOC) [file pone.0067313.s005.doc]

**Table S3.**

**Two-sample Kolmogorov–Smirnov test on length for the 3 sets of 5’UTRs**

|  | D | P-value | H0 (P>0.05) |
| --- | --- | --- | --- |
| Pos. vs neg. | 0.0649 | 2.33E-01 | accept |
| Pos. vs neu. | 0.0424 | 2.26E-01 | accept |
| Neg. vs neu. | 0.0497 | 2.45E-01 | accept |
