# Supplementary material for: BRCA1-Dependent Translational Regulation in Breast Cancer Cells
Source: PLoS One. 2013 Jun 21;8(6):e67313. doi: 10.1371/journal.pone.0067313 (PMC3689694; doi:10.1371/journal.pone.0067313)
Supplement: Table S4 — Statistical parameters on length for the 3 sets of 5′UTRs (DOC) [file pone.0067313.s006.doc]

**Table S4.**

**Statistical parameters on length for the 3 sets of 5’UTRs**

|  | Positive | Negative | Neutral |
| --- | --- | --- | --- |
| Mean | 367.10 | 332.24 | 336.17 |
| Median | 257.50 | 267.00 | 254.00 |
| Sandard dev. | 406.44 | 296.74 | 345.60 |
| Min | 9.00 | 23.00 | 9.00 |
| Max | 4752.00 | 3011.00 | 8869.00 |
